# 17β-Estradiol Treatment Attenuates Neurogenesis Damage and Improves Behavior Performance After Ketamine Exposure in Neonatal Rats

**DOI:** 10.3389/fncel.2019.00251

**Published:** 2019-06-13

**Authors:** Weisong Li, Huixian Li, Haidong Wei, Yang Lu, Shan Lei, Juan Zheng, Haixia Lu, Xinlin Chen, Yong Liu, Pengbo Zhang

**Affiliations:** ^1^Department of Anesthesiology, The Second Affiliated Hospital of Xi’an Jiaotong University, Xi’an, China; ^2^Institute of Neurobiology, National Key Academic Subject, Physiology of Xi’an Jiaotong University, Xi’an, China

**Keywords:** neural stem cells, ketamine, neurotoxicity, 17β-estradiol, p-GSK-3β

## Abstract

Ketamine exposure disturbed normal neurogenesis in the developing brain and resulted in subsequent neurocognitive deficits. 17β-estradiol provides robust neuroprotection in a variety of brain injury models in animals of both sexes and attenuates neurodegeneration induced by anesthesia agents. In the present study, we aimed to investigate whether 17β-estradiol could attenuate neonatal ketamine exposure-disturbed neurogenesis and behavioral performance. We treated 7-day-old (Postnatal day 7, PND 7) Sprague-Dawley rats and neural stem cells (NSCs) with either normal saline, ketamine, or 17β-estradiol before/after ketamine exposure, respectively. At PND 14, the rats were decapitated to detect neurogenesis in the subventricular zone (SVZ) and subgranular zone (SGZ) of the hippocampus by immunofluorescence staining. The proliferation, neuronal differentiation, and apoptosis of NSCs were assessed by immunohistochemistry method and TUNEL assay, respectively. The protein levels of cleaved caspase-3 *in vivo* in addition to GSK-3β and p-GSK-3β *in vitro* were examined by western blotting. Spatial learning and memory abilities were assessed by Morris water maze (MWM) test at PND 42–47. Ketamine exposure decreased cell proliferation in the SVZ and SGZ, inhibited NSC proliferation and neuronal differentiation, promoted NSC apoptosis and led to adult cognitive deficits. Furthermore, ketamine increased cleaved caspase-3 *in vivo* and decreased the expression of p-GSK-3β *in vitro*. Treatment with 17β-estradiol could attenuate ketamine-induced changes both *in vivo* and *in vitro*. For the first time we showed that 17β-estradiol alleviated ketamine-induced neurogenesis inhibition and cognitive dysfunction in the developing rat brain. Moreover, the protection of 17β-estradiol was associated with GSK-3β.

## Introduction

Brain growth spurt (BGS) is critical for the normal development of the central nervous system. Substantial neurogenesis occurs in this period, which is characterized by abundant neural stem cell (NSC) changes including cell proliferation, differentiation, migration, and connection of neural cells (Muramatsu et al., 2007). In rodents, this period lasts from birth to the first 2 weeks of life (Ponten et al., 2012). Since the developing brain is vulnerable to exogenous substrates during BGS, toxic insults may induce functional impairment in learning and memory abilities in adulthood (Eriksson, 1997; Eriksson et al., 2000). It was speculated that neurogenesis damage at an early age would cause cognitive impairment (Kang et al., 2017).

Ketamine is a non-competitive blocker of N-methyl-D-aspartate (NMDA) receptor and is commonly used in pediatric anesthesia. Recent studies have shown that ketamine inhibits NSC proliferation and disturbs normal neurogenesis (Scallet et al., 2004; Lu et al., 2017), causes neuroapoptosis and neurodegeneration in the developing brain, which may ultimately lead to long-term neurocognitive and memory dysfunctions (Paule et al., 2011; Sabbagh et al., 2012). Although some underlying molecular signals such as the PKC/ERK1/2 pathway, reactive oxygen species-mediated mitochondria dysfunction, and glycogen synthase kinase 3β were speculated to be involved in pathophysiological abnormality induced by ketamine exposure (Huang et al., 2012; Bai et al., 2013; Liu et al., 2013), specific adjunctive therapy aiming to mitigate these negative effect of ketamine is still lacking.

17β-estradiol is a principal female hormone, which provides robust neuroprotection in many brain injury models in both sexes and attenuates neurodegeneration induced by anesthesia agents (McCullough and Hurn, 2003; Li et al., 2014). 17β-estradiol also plays a role as a potent modulator for physiological neurogenesis. NSCs derived from embryos and adults both express estrogen receptor a (ERa) and estrogen receptor b (ERb) (Brännvall et al., 2002). 17β-estradiol not only promotes the proliferation and neuronal differentiation of embryonic NSCs (Brännvall et al., 2002; Isgor and Watson, 2005; Kishi et al., 2005), but also regulates the migration of embryonic neuroblasts via ERb (Wang et al., 2003). 17β-estradiol also enhances axonal sprouting, synaptic transmission, and post-stroke neurogenesis (Garcia-Segura et al., 2001; Zheng et al., 2012). However, whether 17β-estradiol administration protects the developing brain from ketamine-caused neurogenesis impairment and improves cognitive dysfunction remains unclear.

As a serine/threonine kinase, glycogen synthase kinase (GSK) 3β plays an important role in multiple fundamental functions of cell in the developing brain, including neurogenesis, apoptosis, cell cycle, cytoskeletal integrity, and axon growth (Hur and Zhou, 2010; Kandimalla et al., 2016). Exposure to ketamine decreased GSK-3β phosphorylation and induced neurotoxicity both in the NSCs and neurons of the neonatal rat brains (Bai et al., 2013; Liu et al., 2013; Huang et al., 2015; Lu et al., 2017). Increasing GSK-3β phosphorylation attenuated ketamine-induced neurogenesis disorder and neural cell injury (Lu et al., 2017, 2018). Interestingly, GSK-3β is also a downstream target of estradiol signaling (Shi et al., 2013; Wu et al., 2014). In the present study, we aimed to figure out whether 17β-estradiol could attenuate neurogenesis damage and cognitive dysfunction induced by ketamine exposure. We also investigated whether the GKS-3β signaling pathway participated in the protective effects of 17β-estradiol on ketamine-induced injury in neurogenesis.

## Materials and Methods

### Animal Protocols

We performed all the experimental protocols according to the National Institutes of Health Guide for the Care and Use of Laboratory Animals (NIH Publications No. 80–23). The animal procedures were approved by the Animal Care and Use Committee of Xi’an Jiaotong University and designed to minimize the number and suffering of rats used. PND 7 and embryonic day 18–19 Sprague-Dawley rats were obtained from Laboratory Animal Centre of Xi’an Jiaotong University.

### Morris Water Maze

The spatial learning and memory function of rats after ketamine exposure were tested by MWM experiments as described in a previous study (Shen et al., 2013). Specifically, PND 42–47 rats (*n* = 10 per group) were trained for place trials and spatial probe tests in a large tank (diameter: 150 cm, depth: 60 cm), which was filled to a depth of 32 cm of warm water (maintained around 25 ± 1°C) and divided into four quadrants. A platform (diameter: 12 cm, height: 30 cm) was placed in the center of the third quadrant (the target) and submerged approximately 2 cm beneath the water surface. We poured milk powder into the water to make the water opaque. We conducted the place trials at PND 42–46 with 4 trials daily at the same time point and performed the probe trials on PND 47 after 5 days’ training. The swimming of rats during the tests was recorded by a video tracking system installed above the tank. In place trials, rats were placed into four quadrants (spaced 20 min apart) to swim freely for a maximum of 120 s. If the rats could not find the platform within 120 s, they were allowed to stay on the platform for 20 s to observe the environment by guiding. The time for rats to reach the platform and swimming speed were recorded. In probes trials, the platform was removed and the rats were put into the first quadrant and allowed to swim for 120 s. The times of rats crossing the original platform were recorded.

### Anesthetic Exposure *in vivo* and Tissue Preparation

The PND 7 rats, weighing 13–18 g, were housed with their mother and maintained at a temperature of 24°C in a 12 h/12 h light/dark cycle with free access to food and water. We assigned the rats randomly into three groups (28 rats from 7 nests in each group, 4 pups per nest): (i) the rats in control group received equal volume of normal saline by intraperitoneal injection as ketamine solution at corresponding time points; (ii) the rats in ketamine group received 40 mg/kg ketamine, diluted in normal saline and administrated by intraperitoneal injection (ketamine, Sigma–Aldrich Inc. St. Louis, MO, United States), the initial injection was considered to be the loading dose, 30% of it was injected at approximately 40 min intervals to maintain the anesthesia for 4 h (Lu et al., 2017); (iii) the rats in the 17β-estradiol group received 17β-estradiol (17β-estradiol, Tocris, Minneapolis, MN, United States; DMSO, Sigma–Aldrich, St Louis, MO, United States) dissolved in dimethylsulfoxide (DMSO) at a concentration of 100 ug/ml, 100 ug/kg 17β-estradiol administered intraperitoneally 8 h, 1 h prior to and 3 h after ketamine’s initial injection (Liu et al., 2007). During anesthesia, all pups were kept on an electric blanket with the temperature set at 36.5 ± 1°C to maintain body temperature and reduce stress. We observed the respiratory rate, skin color, and body movement of rats carefully and tested the voluntary movement by clamping the pup tails. Pulse oxygen saturation (SpO2) was detected by attaching the infant pulse oximetry probes to the rat abdomen. After the anesthesia, the pups received BrdU (50 mg/kg, intraperitoneal injection) every 24 h for 7 consecutive days. On PND 14, the rats were decapitated and the brain tissues of SVZ and SGZ were harvested to detect neurogenesis.

At 12 h after anesthesia, rat pups (*n* = 6 per group, captured randomly) were sacrificed by decapitation. Both the brain tissue from SVZ and SGZ were isolated immediately on ice and the stored at −80°C until use for western blotting. The rats (*n* = 6 per group, captured randomly) were sacrificed and perfused transcardially with 0.9% saline 7 days after anesthesia, followed by cold 4% paraformaldehyde in PBS. Then the harvested brains were postfixed in 4% paraformaldehyde overnight at 4°C and dehydrated in 30% sucrose solution for 3–4 days, as we described previously (Lu et al., 2017). The brain tissue from bregma +0.2 mm to bregma −6.0 mm was the region of interest, which were cut into 16 μm coronary tissue slices by freezing microtome (SLEE, Germany). These brain slices were collected and used for future immunohistochemistry staining. The rest of the rat pups (*n* = 10 per group) were bred for behavior study at adulthood.

### Immunohistochemistry

Immunohistochemistry was used to evaluate NSC proliferation in SVZ and SGZ by BrdU staining. Firstly, the brain slices were incubated with 2 N HCl for 30 min to denaturate the DNA at 37°C. After being incubated with 0.1 mol L^−1^ boric acid (pH 8.5) for 10 min at room temperature followed by three times washing with 0.1 M PBS, the slices were blocked by 2% goat serum and 0.3% Triton X-100 for 2 h at room temperature, then incubated with the mouse monoclonal anti-BrdU antibody (1:200, Abcam, United Kingdom) at 4°C overnight. The next day, after three washings with 0.1 M PBS, the slices were incubated with tetramethyl rhodamine isothiocyanate (TRITC)-conjugated secondary antibodies for 2 h at room temperature. BrdU-positive cells were counted within defined regions of interest in the SVZ and SGZ. In total, the mean numbers of BrdU-positive cells of six brain slices for each rat, spaced approximately 200 μm apart, were examined by the observer blindly. For each slice, five regions were captured by fluorescence microscopy (BX51, Olympus, Tokyo, Japan), and the planar area enclosed by each region was 50 × 50 μm. The edges of the captured regions were defined according to structural details to ensure the fields did not overlap (Zhang et al., 2009). The density of positive cells was presented as the total number of BrdU-positive cells in the SVZ and SGZ.

### NSC Culture

Primary cultured NSCs were obtained from the cortex of rat at embryonic day 18–19 under sterile conditions. Briefly, the forebrain portion was isolated and placed in ice-cold Hank’s solution (without Mg^2+^ and Ca^2+^, Gibco, Carlsbad, CA, United States). The tissues were then dissociated and triturated mechanically by a fire-polished Pasteur pipette softly. After centrifugation, the isolated cells were collected and re-suspended in free-serum DMEM/F12 medium (Gibco, Carlsbad, CA, United States) which was supplemented with 2% B27 (Gibco, Carlsbad, CA, United States), 20 ng/ml EGF (Gibco, Carlsbad, CA, United States), 20 ng/ml bFGF (Gibco, Carlsbad, CA, United States), and 100 U/ml penicillin and phytomycin. Cells were cultured for 7 days to form enough neurospheres and then passaged at a density of 2 × 10^5^ cells/ml followed by collection and dissociation as previously described by Reynolds and Weiss (Reynolds et al., 1992). Half of the medium was changed every 3 days. After the second passage, the cells were ready for future experiments. For identification assessment, the cells after passage were seeded onto 100 μg/mL poly-L-lysine-coated coverslips and cultured in differentiating medium that contained 100× N2 supplement, 100× B27 supplement, and 1% fetal bovine serum (FBS, Gibco, Carlsbad, CA, United States) in DMEM/F12 (without b-FGF) for 7 days.

### Drug Exposure and Neurogenesis Analysis *in vitro*

The cells were assigned to the following groups: control group, ketamine group, and 17β-estradiol group. No drug treatment was added to the control group. NSCs in the ketamine group were exposed to 100 μM ketamine for 24 h. NSCs in the 17β-estradiol group were pretreated with 17β-estradiol (100 nM) for 30 min and then 100 μM of ketamine was added to the culture medium for 24 h. For proliferative analysis, NSCs were seeded on cover slips which were pre-coated with 100 μg/mL poly-L-lysine and incubated with BrdU for the last 4 h. Following being fixed with 4% paraformaldehyde, the cells were stained with BrdU antibody (1:200, Abcam, United Kingdom) and DAPI. As for neuronal differentiation analysis, after being exposed to ketamine with or without 17β-estradiol for 24 h, the cells were seeded on cover slips which were pre-coated with 100 μg/mL poly-L-lysine and incubated with differentiating medium for 7 days, then the cells were harvested for immunohistochemical staining. The cells were labeled with β-tubulin III antibody (1:500; Sigma-Aldrich Inc. St. Louis, MO, United States). Briefly, 5–7 randomly selected fields were captured in each coverslip, and the numbers of β-tubulin III-positive cells were counted (at least 200 cells per test case). Data were collected from three independent experiments.

### Cell Apoptosis Test

We used terminal dUTP nick-end Labeling (TUNEL) assay to detect cell apoptosis. Briefly, after passage, the dissociated cells were exposed to ketamine with or without 17β-estradiol for 24 h. After the treatments, cells were fixed with 4% paraformaldehyde for 15 min. The TUNEL assay was performed according to the instruction of *in situ* Cell Death Detection Kit (Roche Inc. Roche, Mannheim, Germany). Data were collected from three independent experiments.

### Western Blot Analysis

Brain tissues from the SVZ and SGZ of rats (*n* = 6 per group) at 12 h after anesthesia and cell cultures at 24 h following drug exposure were subjected to Western blot analyses as described in our previous studies (Lu et al., 2017). Briefly, the tissues were lysed by RIPA lysis buffer with protease and phosphatase inhibitors. The lysates were homogenized with an electric homogenizer and maintained on ice for 15 min. After being centrifuged for 15 min at 14000 rpm at 4°C, the supernatant was aspirated and the resulting lysates were placed in a new tube. We used the BCA protein assay kit to examine the protein concentrations. Bovine serum albumin (BSA) was used as a standard. An equal amount of the resulting lysate was resolved by sodium dodecyl sulfate-polyacrylamide gel and the separated proteins were transferred to polyvinylidene fluoride membranes. After being blocked for 1 h at room temperature, the membranes were then incubated with appropriate dilutions of primary antibodies at 4°C overnight. The used antibodies included anti-caspase-3 (cleaved, 17 KDa, 1:1000, Cell Signal Technology Inc. Beverly, MA, United States), anti-phosophorylated GSK-3β (p-GSK-3β, 1:1000, Cell Signal Technology Inc. Beverly, MA, United States), anti-GSK-3β (1:1000, Cell Signal Technology Inc. Beverly, MA, United States), and anti-β-actin (1:1000, Cell Signal Technology Inc., Beverly, MA, United States). The following day, the membranes were incubated with horseradish peroxidase-conjugated secondary antibodies (goat anti-rabbit or anti-mouse) for 2 h at room temperature. After being enhanced by chemiluminescence (ECL), the signals were then exposed to X-ray films. Each band in the Western blot represented an independent experiment and at least three independent experiments were conducted. Data were expressed as the ratio to optical density (OD) values of the corresponding controls. The Western blots were quantified as described in our previous study.

### Statistical Analysis

Data obtained from the study were presented as mean ± SEM. Every data point represented a mean for each animal in a single case. SigmaPlot 12.0 was used for all statistical analysis. Data were tested and then confirmed with normality and equal variance criteria. A one-way analysis of variance (ANOVA) following the *post hoc* Holm-Sidak method was used to analyze the differences among different groups. A two-tailed probability value *P* < 0.05 was considered statistically significant.

## Results

### 17β-Estradiol Improved Ketamine-Induced Decline of Learning and Memory

**FIGURE 1:**
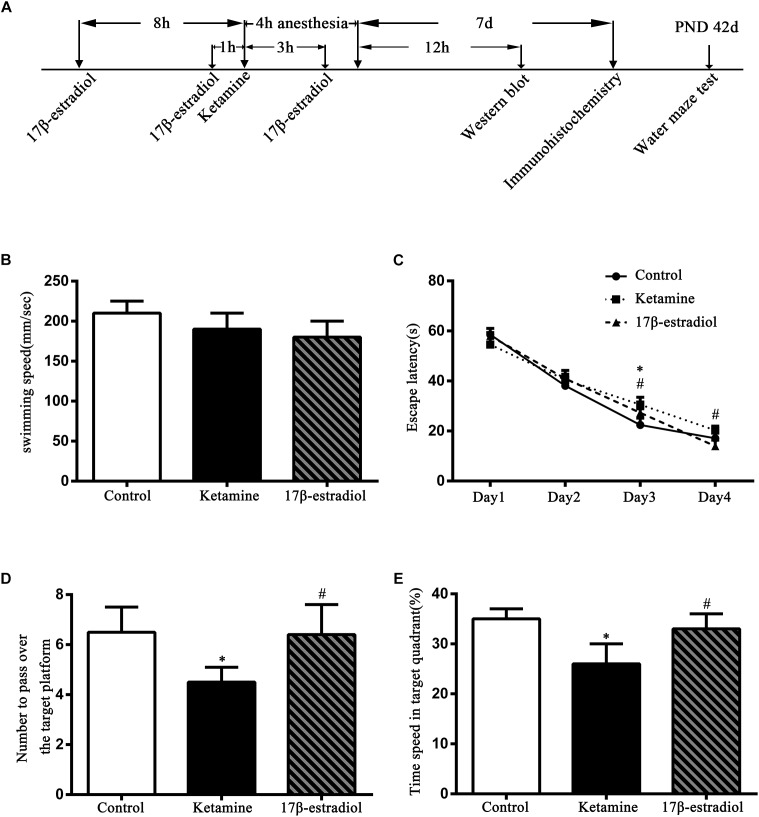
Diagram of the study and results of Morris water maze trials. **(A)** The diagram of the timeline of the study. **(B)** Swimming speed comparison between three groups during the training. There was no difference among three groups. **(C)** Comparison of the latency to reach the hidden platform. ^∗^*p* < 0.05 compared with control group, ^#^*p* < 0.05 compared with ketamine group. **(D)** Comparison of the numbers to pass over the target platform. ^∗^*p* < 0.05 compared with control group, ^#^*p* < 0.05 compared with ketamine group. **(E)** Comparison of the time spent in target quadrant. ^∗^*p* < 0.05 compared with control group, ^#^*p* < 0.05 compared with ketamine group. Data are present as the means ± SEM. *n* = 6 in each group.

The MWM test results are shown in Figure 1. There was no significant difference in the swimming speed among the control, ketamine, and 17β-estradiol groups [Figure 1B; *F*(2,36) = 8.956; *p* > 0.05]. When compared to the control group, the escape latency was increased in the ketamine group on trial day 3 [Figure 1C; *F*(2,48) = 50.65; *p* < 0.05]. However, when compared to the ketamine group, the escape latency was decreased in the 17β-estradiol group on both trial days 3 and 4 [Figure 1C; *F*(2,48) = 62.35; *p* < 0.05]. The times to pass over the target platform and time spent in target area are shown in Figure 1D,E. Compared with the control group, rats in the ketamine group had less time to pass over the target platform and spent less time in the target area [*F*(2,72) = 89.64; *p* < 0.05]. In contrast, compared with the ketamine group, rats in the 17β-estradiol group passed over the target platform more frequently and spent more time in the target quadrant [*F*(2,60) = 70.35; *p* < 0.05]. Collectively, these results showed that ketamine exposure in neonatal rats would induce cognitive impairment in adulthood and that pretreatment with 17β-estradiol could attenuate this defect.

### 17β-Estradiol Enhanced Proliferation and Reduced Apoptosis of Cells in the SVZ and SGZ Following Ketamine Exposure

**FIGURE 2:**
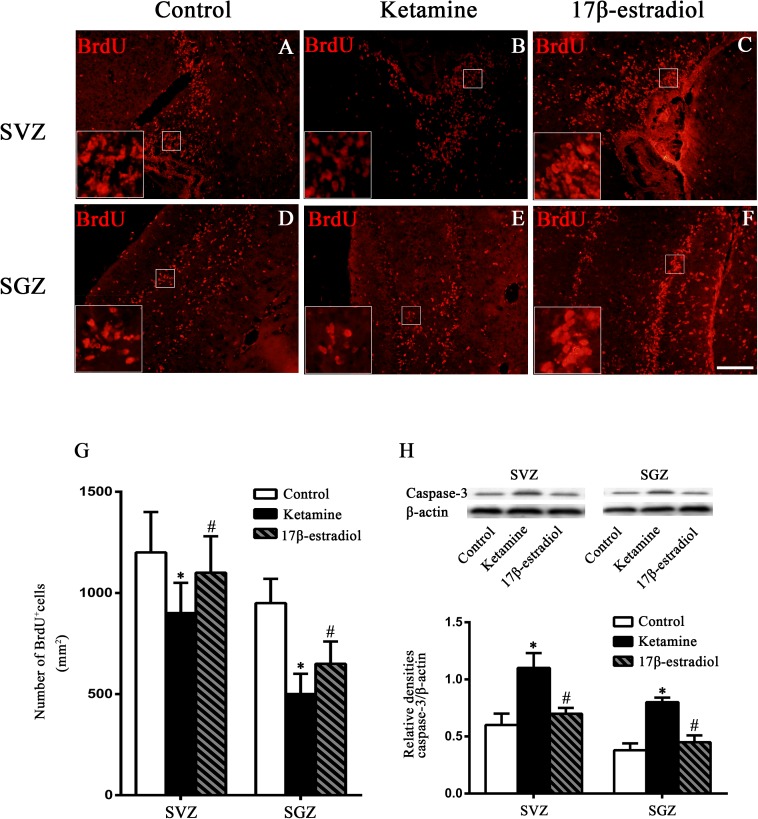
The proliferative changes in the SVZ and SGZ. **(A–F)** Representative images of BrdU immunoreactive cells (red) in the SVZ and SGZ 7 days after anesthesia. Scale bar = 100 μm. **(G)** Quantification of BrdU-positive cells following different treatment. ^∗^*p* < 0.05 compared with control group, ^#^*p* < 0.05 compared with ketamine group. **(H)** The expression and quantification of caspase-3 by western blotting in the SVZ and SGZ 12 h after anesthesia. ^∗^*p* < 0.05 compared with control group, ^#^*p* < 0.05 compared with ketamine group. Data are presented as the means ± SEM. *n* = 6 in each group.

BrdU incorporation was used to assess cell proliferation. As shown in Figure 2, BrdU-positive cells were distributed in the SVZ and SGZ among all groups. Less BrdU-positive cells were found in the SVZ and SGZ of the ketamine group. Increased BrdU-positive cells were detected in both regions of the control and 17β-estradiol groups. Quantitative analysis showed that the number of BrdU-positive cells in SVZ and in SGZ of ketamine group declined significantly when compared to the control group [*F*(2,36) = 31.97; *p* < 0.01, in SVZ and *F*(2,60) = 6.031; *p* < 0.01 in SGZ]. The number of BrdU-positive cells in the 17β-estradiol group was significantly higher than that of the ketamine group both in SVZ and in SGZ [*F*(2,36) = 31.97; *p* < 0.05 in SVZ and *F*(2,60) = 6.031; *p* < 0.05 in SGZ], respectively. However, it was less than that of the control group in SVZ [*F*(2,36) = 31.97; *p* < 0.05], but not in SGZ [*F*(2,60) = 6.031; *p* > 0.05] (Figure 2G).

Considering the important role of cleaved caspase-3 in cell apoptosis (Moosavi et al., 2012), the protein levels of cleaved caspase-3 in the SVZ and SGZ 12 h after ketamine exposure were examined by Western blotting. When compared to the control group, where the levels of cleaved caspase-3 were relatively low, ketamine exposure increased the cleaved caspase-3 expressions in both regions [*F*(2,6) = 8.627; *p* < 0.05, in SVZ and *F*(2,6) = 9.742; *p* < 0.05 in SGZ]. However, pretreatment with 17β-estradiol decreased protein expressions of cleaved caspase-3 in the SVZ and SGZ of neonatal rats after ketamine exposure[*F*(2,6) = 8.627; *p* < 0.05, in SVZ and *F*(2,6) = 9.742; *p* < 0.05 in SGZ] (Figure 2H).

### 17β-Estradiol Rescued Proliferation and Apoptosis of NSCs Following Ketamine Exposure While It Reversed the Decrease of Neuron Production Induced by Ketamine

**FIGURE 3:**
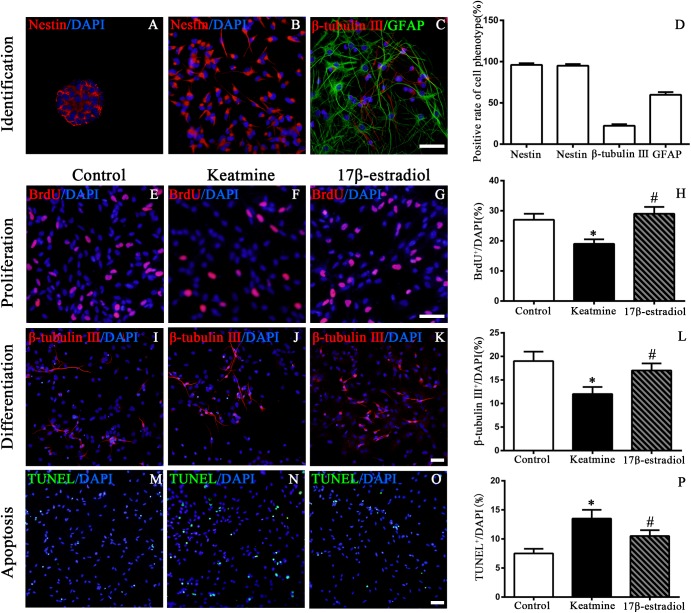
Identification of cultured cells and the proliferation, differentiation as well as apoptosis of NSCs following different treatment. **(A)** Images of nestin (red) immunoreactive neurosphere. **(B)** Images of nestin (red) immunoreactive NSCs. **(C)** Images of NSCs differentiating into neurons (red) and astrocyte (green). Scale bar = 100 μm. **(D)** Rate of specific cellular phenotype to total cells (DAPI, blue). **(E–G)** Representative images of BrdU immunoreactive cells (red) in control, ketamine, and 17β-estradiol group, respectively. Scale bar = 100 μm. **(H)** Quantification of BrdU-positive cells following different treatment. ^∗^*p* < 0.05 compared with control group, ^#^*p* < 0.05 compared with ketamine group. **(I–K)** Representative images of β-tubulinimmunoreactive cells (red) in control, ketamine, and 17β-estradiol group, respectively. Scale bar = 50 μm. **(L)** Quantification of β-tubulin III -positive cells following different treatment. ^∗^*p* < 0.05 compared with control group, ^#^*p* < 0.05 compared with ketamine group. **(M–O)** Representative images of TUNEL immunoreactive cells (green) in control, ketamine, and 17β-estradiol groups, respectively. Scale bar = 50 μm. **(P)** Quantification of TUNEL-positive cells following different treatment. ^∗^*p* < 0.05 compared with control group, ^#^*p* < 0.05 compared with ketamine group. Data were collected from three independent experiments and are presented as the means ± SEM.

To identify the characteristics of cultured cells, we first stained the cells with NSC marker nestin 3 days after seeding. The results showed that both in suspension and adherent culture most of the cells were nestin-positive. The percentages of nestin-positive cells were 96.0 ± 2.1 and 95.0 ± 2.0%, respectively (Figure 3A,B). After incubation with differentiating medium for 7 days, the cultured cells expressed neuronal marker β-tubulin III (22.3 ± 1.9%, Figure 3C,D) and astrocytes marker glial fibrillary acidic protein (GFAP) (59.8 ± 3.2%, Figure 3C,D). Taken together, these data suggested that the cells used in this study were NSCs.

To examine the proliferation of NSCs *in vitro*, BrdU incorporation method was used (Figure 3E–G). It was shown that the number of BrdU-positive cells was decreased after exposure to ketamine for 24 h when compared to the control group [*F*(2,42) = 148.6; *p* < 0.05]. However, pretreatment with 17β-estradiol increased the number of BrdU-positive cells following ketamine exposure. There was no significant difference between the 17β-estradiol group and the control group [*F*(2,42) = 148.6; *p* > 0.05] (Figure 3H). These findings indicated that 17β-estradiol rescued the proliferation of NSCs exposed to ketamine.

Neuronal differentiation of NSCs was assessed by immunofluorescence staining of β-tubulin III (Figure 3I–K). Ketamine exposure for 24h decreased the percentage of β-tubulin III-positive cells, indicating ketamine inhibited neuronal production from NSCs. However, co-administration with 17β-estradiol reversed the effects of ketamine on neuronal production [*F*(2,42) = 60.36; *p* < 0.05] (Figure 3L, *p* < 0.05). There was no significant difference between the 17β-estradiol group and the control group [*F*(2,42) = 60.36; *p* > 0.05]. These findings indicated that 17β-estradiol rescued the decrease of neuronal production from NSCs exposed to ketamine.

Next, we used TUNEL staining to assess the apoptosis of NSCs, aiming to investigate whether 17β-estradiol could reduce NSC apoptosis induced by ketamine exposure (Figure 3M–O). After ketamine exposure, the number of TUNEL positive cells was obviously increased when compared with the control condition. However, pretreatment with 17β-estradiol significantly reduced NSC apoptosis induced by ketamine exposure [*F*(2,42) = 27.81; *p* < 0.05] (Figure 3P).

**FIGURE 4:**
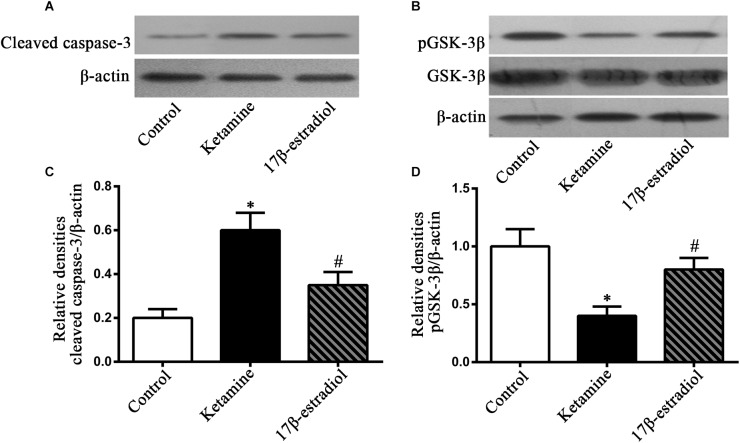
Detection of cleaved caspase-3 and pGSK-3β by western blotting. **(A,B)** Representative images of western blotting analysis of the cleaved caspase-3 and pGSK-3β in NSCs 24 h following drug exposure. **(C,D)** Quantification of cleaved caspase-3 and pGSK-3β expression normalized to β-actin following different treatment. ^∗^*p* < 0.05 compared with control group, ^#^*p* < 0.05 compared with ketamine group. Data were collected from three independent experiments and are expressed as the ratio to optical density (OD) values of the corresponding controls.

We used western blotting to determine related molecules involved in ketamine-induced damage and 17β-estradiol-elicited neuroprotection (Figure 4A,B). The results showed that ketamine exposure for 24h increased cleaved caspase-3 expression [*F*(2,24) = 76.59; *p* < 0.05] and decreased p-GSK-3β expression [*F*(2,42) = 43.28; *p* < 0.05] in NSCs compared to the control. Pretreatment with 17β-estradiol decreased the levels of cleaved caspase-3 [*F*(2,24) = 76.59; *p* < 0.05] and prevented the deregulation of p-GSK-3β expressions [*F*(2,42) = 43.28; *p* < 0.05] in NSCs exposed to ketamine for 24 h. However, there were no differences in the protein expressions of p-GSK-3β [*F*(2,42) = 43.28; *p* > 0.05] and cleaved caspase-3 [*F*(2,24) = 76.59; *p* > 0.05] between the control group and 17β-estradiol group (Figure 4C,D). These findings suggest that GSK-3β and caspase-3 were involved in ketamine-induced apoptosis and 17β-estradiol elicited protection.

## Discussion

In the present study, our data showed that treatment with 17β-estradiol improved neonatal ketamine exposure-induced cognitive deficits and mitigated ketamine-caused changes in cellular proliferation and apoptosis in the SVZ and SGZ. Treatment with 17β-estradiol rescued neurogenesis and reduced apoptosis of NSCs exposed to ketamine *in vitro*, in which GSK-3β might play a role in 17β-estradiol-elicited protection.

The toxicity of general anesthesia on the developing brain has raised concern in recent years. It is a consensus that repeated exposure to general anesthetic before 3 years of age is harmful to the developing brain (Hu et al., 2017). Ketamine is a commonly used anesthetic in pediatric clinics. It has been proven that ketamine can lead to long-lasting cognitive impairments in rodent and primate models (Fredriksson et al., 2007; Paule et al., 2011; Huang et al., 2012; Moosavi et al., 2012; Zhao et al., 2014). Unfortunately, there has been no safe and effective measure to prevent this deficit until now. To evaluate whether 17β-estradiol could improve the learning and memory ability of adult rats that were subjected to ketamine exposure during the neonatal stage, the Morris water maze was used in this study. The rats in the 17β-estradiol group passed over the target platform more frequently and spent more time in the target area compared with the ketamine group, suggesting that 17β-estradiol ameliorates long-lasting cognitive dysfunction in rodents who received ketamine in the early stage of life. Consistently, Li et al. also reported that 17β-estradiol attenuated long-term cognitive impairments in developing rats though they used higher doses of ketamine and 17β-estradiol and performed the Morris water maze at an older age than that used in this study (Li et al., 2014). These findings indicate a potential strategy for prevention of ketamine-induced neurocognitive deficits.

Many studies showed that ketamine caused neuroapoptosis and neurodegeneration in the developing brain, which may finally induce learning and memory disabilities in adults (Fredriksson et al., 2007; Paule et al., 2011; Huang et al., 2012; Moosavi et al., 2012; Sabbagh et al., 2012). However, few studies reported the effect of ketamine on neurogenesis and its long-term outcome *in vivo*. In mammals, new neurons are generated continuously to certain brain areas throughout life. These neurons are differentiated from NSCs located primarily in the SVZ and SGZ. Proliferation and/or survival of NSCs are the basic events in neurogenesis. Considering the significance of neurogenesis during the BGS period, we evaluated the NSC proliferation and survival using BrdU labeling and apoptosis analysis. It was shown that ketamine inhibited the cell proliferation in the neurogenesis regions of neonatal rats, indicating that neonatal ketamine exposure might impair neurogenesis. This is consistent with previous studies (Huang et al., 2015, 2016; Dong et al., 2016). Interestingly, we observed that 17β-estradiol alleviates cellular proliferating changes induced by ketamine in neurogenesis regions of neonatal rats, indicating that 17β-estradiol enhanced NSC proliferation following ketamine exposure during brain development, which is a benefit for neurogenesis. Furthermore, our *in vitro* study demonstrated that 17β-estradiol rescued proliferation and neuronal production of NSCs exposed to ketamine. Whether anesthetic-induced neurogenesis inhibition contributes to any disabilities in learning and memory functions remains unknown. Our recent work showed that restoration of neurogenesis in SVZ and SGZ in neonatal rats could improve the neonatal ketamine exposure-induced adult spatial learning and memory deficits (Lu et al., 2017). In this study, treatment with 17β-estradiol mitigated ketamine-induced changes in neurogenesis and cognitive deficits. Li et al. reported that ketamine induced neuroapoptosis in the prefrontal cortex of the developing brain and caused long-term cognitive dysfunction in adulthood, and treatment with 17β-estradiol attenuated ketamine-induced damages (Li et al., 2014). Our previous work showed that ketamine exposure did not increase the rate of TUNEL-positive cells in the frontal cortex (Lu et al., 2017). The discrepancy might be due to dose of drugs, regions of interest and endpoints of observation. Collectively, our findings supported a causal link between neurogenesis damage and cognitive dysfunction in neurodevelopmental toxicity of anesthetics.

Apoptosis is a critical procedure during the development of the neural system, which occurs at various developmental stages from neurogenesis to adulthood (Hara et al., 2018). However, exogenous insult-induced apoptosis had a lasting impact on neurogenesis in the developing brain (Sokolowski et al., 2013). Maintenance of the homeostasis of neurogenesis provides basis for normal brain structure and function. As an antagonist of non-competitive NMDA receptor, ketamine induced NSC apoptosis by activation of GSK-3β (Lu et al., 2018). It was reported that ketamine induced neuroapoptosis in the prefrontal cortex accompanied by the downregulation of 17β-estradiol, BDNF, and p-Akt in neonatal rats, and treatment with 17β-estradiol attenuated ketamine-induced injuries (Li et al., 2014). We found that treatment with 17β-estradiol decreased NSC apoptosis and increased p-GSK-3β levels, indicating that 17β-estradiol decreased NSC apoptosis by inactivation of GSK-3β. Further study needs to be done to elucidate how ketamine or 17β-estradiol affect GSK-3β phosphorylation in the NSCs. NSCs express ERa and ERb (Brännvall et al., 2002). 17β-estradiol promotes NSC proliferation and differentiation into neurons (Brännvall et al., 2002; Isgor and Watson, 2005; Kishi et al., 2005). In the present study, whether the protection of 17β-estradiol on ketamine-induced neurogenesis damage is merely due to the increase in NSC proliferation and neuronal differentiation or the decrease in NSC apoptosis needs to be further studied.

Although some important discoveries were revealed by these studies, it is worth emphasizing that several limitations exist. Firstly, NSC differentiation, neuronal apoptosis, or migration in the developing brain were not detected because our main aim for this study was to observe whether anesthesia doses of ketamine could inhibit NSC proliferation, induce NSC apoptosis in neonatal rats, and cause cognitive deficits in adults as well as whether treatment with 17β-estradiol could attenuate these changes. Further study should be done to reveal NSC differentiation, neuronal apoptosis, or migration in the developing brain exposed to ketamine with or without 17β-estradiol. Secondly, only *in vitro* neuronal differentiation of NSCs was investigated in the study. Later studies should select more indexes to detect differentiation of NSCs *in vitro*. Thirdly, GSK-3β is the target of numerous molecules, but we did not investigate the upstream and downstream signal of GSK-3β caused by ketamine or 17β-estradiol, and further studies are needed.

## Conclusion

In conclusion, using a model of neurotoxicity following ketamine exposure to neonatal rats, we found that ketamine exposure increased apoptosis and decreased proliferation of cells in the SVZ and SGZ, leading to a decline in spatial learning and memory abilities in adulthood. Administration of 17β-estradiol enhanced neurogenesis by decreasing apoptosis and increasing proliferation of NSCs. Furthermore, GSK-3β might be an important molecule that is involved in this process. The present findings provide an experimental basis for the use of 17β-estradiol as a therapeutic drug against the development neurotoxicity of ketamine. Clinically, the results suggest 17β-estradiol may help to prevent ketamine-induced developmental neurotoxicity and GSK-3β may become a molecular target for its treatment. Nevertheless, a much work still needs to be done before clinical use, because a more specific treatment target should be developed and translated to humans.

## Ethics Statement

This study was carried out in accordance with the recommendations of the National Institutes of Health Guide for the Care and Use of Laboratory Animals (NIH Publications No. 80–23). The protocol was approved by the Animal Care and Use Committee of Xi’an Jiaotong University.

## Author Contributions

PZ, WL, and HuL conceived and designed the experiments. WL, HuL, YaL, SL, and JZ performed the experiments. HW, SL, and XC analyzed the data. HaL and YoL contributed with the Materials and Methods, and critically revised the manuscript. All authors wrote the manuscript.

## Conflict of Interest Statement

The authors declare that the research was conducted in the absence of any commercial or financial relationships that could be construed as a potential conflict of interest.
